# Association of Clinical Features with Spike Glycoprotein Mutations in Iranian COVID-19 Patients

**DOI:** 10.3390/jcm11216315

**Published:** 2022-10-26

**Authors:** Shahrzad Ahangarzadeh, Alireza Yousefi, Mohammad Mehdi Ranjbar, Arezou Dabiri, Atefeh Zarepour, Mahmoud Sadeghi, Elham Heidari, Fariba Mazrui, Majid Hosseinzadeh, Behrooz Ataei, Ali Zarrabi, Laleh Shariati, Shaghayegh Haghjooy Javanmard

**Affiliations:** 1 Infectious Diseases and Tropical Medicine Research Center, Isfahan University of Medical Sciences, Isfahan 81746-73461, Iran; 2 Razi Vaccine and Serum Research Institute, Agricultural Research, Education, and Extension Organization (AREEO), Karaj 31585-854, Iran; 3 Applied Physiology Research Center, Cardiovascular Research Institute, Isfahan University of Medical Sciences, Isfahan 81746-73461, Iran; 4 Department of Biomedical Engineering, Faculty of Engineering and Natural Sciences, Istinye University, Istanbul 34396, Turkey; 5 Health Vice Chancellery of Isfahan Medical University, Isfahan University of Medical Sciences, Isfahan 81656-47194, Iran; 6 Department of Genetics and Molecular Biology, School of Medicine, Isfahan University of Medical Sciences, Isfahan 81746-73461, Iran; 7 Nosocomial Infection Research Center, Isfahan University of Medical Sciences, Isfahan 31379-23929, Iran; 8 Department of Biomaterials, Nanotechnology and Tissue Engineering, School of Advanced Technologies in Medicine, Isfahan University of Medical Sciences, Isfahan 81746-73461, Iran; 9 Biosensor Research Center, School of Advanced Technologies in Medicine, Isfahan University of Medical Sciences, Isfahan 81746-73461, Iran

**Keywords:** COVID-19, clinical symptoms, spike glycoprotein, mutation, D614G

## Abstract

Background: Mutations in spike glycoprotein, a critical protein of SARS-CoV-2, could directly impact pathogenicity and virulence. The D614G mutation, a non-synonymous mutation at position 614 of the spike glycoprotein, is a predominant variant circulating worldwide. This study investigated the occurrence of mutations in the crucial zone of the spike gene and the association of clinical symptoms with spike mutations in isolated viruses from Iranian patients infected with SARS-CoV-2 during the second and third waves of the COVID-19 epidemic in Isfahan, the third-largest city in Iran. Methods: The extracted RNA from 60 nasopharyngeal samples of COVID-19 patients were subjected to cDNA synthesis and RT-PCR (in three overlapping fragments). Each patient’s reverse transcriptase polymerase chain reaction (RT-PCR) products were assembled and sequenced. Information and clinical features of all sixty patients were collected, summarized, and analyzed using the GENMOD procedure of SAS 9.4. Results: Analysis of 60 assembled sequences identified nine nonsynonymous mutations. The D614G mutation has the highest frequency among the amino acid changes. In our study, in 31 patients (51.66%), D614G mutation was determined. For all the studied symptoms, no significant relationship was observed with the incidence of D614G mutation. Conclusions: D614G, a common mutation among several of the variants of SARS-CoV-2, had the highest frequency among the studied sequences and its frequency increased significantly in the samples of the third wave compared to the samples of the second wave of the disease.

## 1. Introduction

A novel strain of coronavirus, SARS-CoV-2, suddenly appeared in Wuhan (China) in December 2019 and has outspread swiftly to the world. On 11 March 2020, WHO announced the rapidly spreading coronavirus disease as COVID-19, the fifth pandemic since the 1918 flu pandemic [1]. SARS-CoV-2 belongs to the betacoronavirus, and its genome encodes four structural proteins by four structural genes, spike (S), envelope (E), membrane (M), and nucleocapsid (N) genes [2]. The host range is controlled by multiple molecular interactions, including receptor interaction. Despite amino acid mutation in some key regions, the structure of the envelope spike (S) protein receptor-binding domain of SARS-CoV-2 is similar to that of SARS-CoV [3]. Immense structural investigations powerfully demonstrate that SARS-CoV-2 may use host receptor angiotensin-converting enzyme 2 (ACE2) to enter the cells [4], the same receptor that helps SARS-CoV to infect the airway epithelium and alveolar type 2 (AT2) pneumocytes, which are pulmonary cells that synthesize pulmonary surfactant [5]. In general, coronavirus’s spike protein comprises two subunits: S1 and S2. The S1 subunit contains the N-terminal domain (NTD), receptor-binding domain (RBD), and subdomain 1 and 2 (SD1/2), which is responsible for binding to ACE2 receptor in target cells, and the S2 domain is responsible for cell membrane fusion during viral infection [6]. Consequently, this protein has an indispensable role in antigen-inducing potent immune response and is the main target for designing and developing vaccines and therapeutics [7]. Moreover, information on SARS-CoV-2 mutations, particularly mutations in surface proteins, such as spike proteins, is essential for estimating virus behavior in drug resistance, immune escape, and pathogenesis [8,9]. The D614G mutation, a non-synonymous mutation at position 614 of Spike glycoprotein, is a predominant variant circulating worldwide [10,11]. Many studies showed that this mutation is associated with higher infectivity [12], higher level of viral load in the upper respiratory tract [13], and higher fatality [14].

Clinical manifestations of 2019-nCoV infection are similar to SARS-CoV. At the onset of the disease, the most prevalent symptoms include fever, dry cough, dyspnea, chest pain, fatigue, and myalgia. Uncommon symptoms include headache, nasal congestion, dizziness, abdominal pain, diarrhea, nausea, and vomiting [15,16]. As the number of patients rises, there are concerns about accumulation of more severe and toxic viral variants. Drug and vaccine therapies can also cause specific and new mutations; therefore, tracing the characteristics of these variants, phylogeny, and at the same time, the demographic characteristics of the disease in different waves of COVID-19, are critical. This study investigated the occurrence and prevalence of mutation models in the crucial zone of the spike gene and the association of clinical symptoms with spike glycoprotein mutations in isolated viruses from Iranian patients infected with SARS-CoV-2 during the second and third waves of the COVID-19 epidemic in Isfahan, the third-largest city in Iran and the eighteenth most populous metropolis in the Middle East.

## 2. Material and Methods

### 2.1. Study Design and Participants

**Figure 1:**
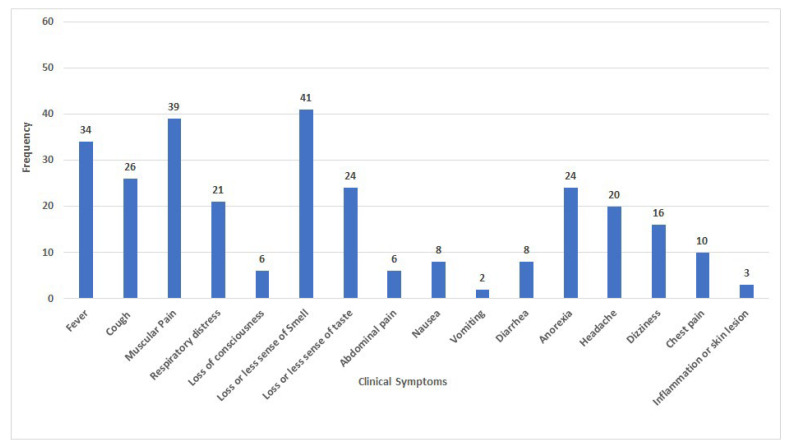
Frequency of clinical symptoms among 60 studied patients.

A total of 60 COVID-19 patients (30 samples belonged to the second wave and 30 to the third wave of the COVID-19 pandemic in the specialized clinics in Isfahan (Iran)) were considered to participate in this monocentric study from August to November 2020 in a prospective study. We chose RNA samples based on previous information of laboratory criteria (real-time PCR). SARS-CoV-2 infection was confirmed by reverse transcriptase real-time PCR. Then we contacted the patient and asked questions according to the questionnaire prepared for the symptoms of the disease. Clinical manifestations such as the presence or absence of fever or chills, shortness of breath, cough and sneezing, sore throat, runny nose, and nausea and vomiting are considered. Figure 1 presents the demographic and clinical characteristics of all study subjects. Also, to evaluate the relationship between the patient’s sex with COVID-19 symptoms, patients were divided in two group: men and women. To investigate the relationship between age and COVID-19 symptoms, patients were divided into two groups according to age: ≤40 years and >40 years.

### 2.2. Reverse Transcription and RT-PCR

Five ng of each RNA sample, confirmed by a real-time PCR test, was used to synthesize the complementary DNA (with random hexamer primer) using the RevertAid First Strand cDNA Synthesis Kit (Biotechrabbit, Hennigsdorf, Germany), according to the manufacturer’s protocol. Three pairs of primers were used to amplify three consecutive fragments of the spike gene sequence. The first fragment was amplified with F1 (TATCTTGGCAAACCACGCGA) and R1 (ACCAGCTGTCCAACCTGAAG) primers; the second fragment was amplified with F2 (CCCTCAGGGTTTTTCGGCTT), and R2 (CTGTGGATCACGGACAGCAT) primers, and the last fragment was amplified with F3 (CCAGCAACTGTTTGTGGACC) and R3 (GTGGCAAAACAGTAAGGCCG) primers. PCR reaction was performed in an Applied Biosystems ^®^ GeneAmp ^®^ PCR System according to the cycling program: 94 °C for 3.5 min, 94 °C for 40 s, 58/59/59.5 °C, respectively, for each fragment’s primer for 45 s, and 72 °C for 2 min, 39 cycles, and a final extension step of 72 °C for 10 min.

### 2.3. Sequencing and Analysis

The final amplified fragments were prepared for sequencing using standard Sanger sequencing technology (GenFanavan Co., Tehran, Iran). To demonstrate and check the sequencing quality, we took advantage of Finch TV and Bioedit software (Geospiza Inc., Seattle, WA, USA). Final sequences were assembled from three overlapping sequence reads of each sample, and the authors submitted 100 assembled sequences (partial S gene sequences) to NCBI GenBank (Supplementary Table S1).

### 2.4. Mutation Detection

A multiple sequence alignment of all 100 sequences was performed to define the single nucleotide polymorphism (SNP) using CLUSTALW and the reference S gene sequence. Mutation at nucleotide and amino acid levels was analyzed using MEGA (Molecular Evolutionary Genetics Analysis Platform) version X software [17]. Finally, the statistical analysis determined the relationship between clinical findings and genetic mutations.

### 2.5. Statistical Analysis

For statistical analysis, each patient’s information was entered into SAS 9.4 (SAS Institute Inc., Cary, NC, USA) and analyzed using the GENMOD procedure. The data were analyzed using descriptive statistics, chi-square tests, independent *t*-tests, and one-way analysis of variance. A *p*-value < 0.05 was accepted as significant.

## 3. Results

### 3.1. Clinical Symptoms

The frequency of clinical features among all sixty patients was summarized. All patients participating in this study exhibited moderate to mild symptoms. As shown in Figure 1, symptoms such as loss of sense of smell, muscular pain, and fever were the most common among the patients.

### 3.2. Identified Single Nucleotide Mutations

Our sequencing results identified nine nonsynonymous mutations in at least five patients, resulting in amino acid changes in spike glycoprotein (Supplementary Table S1). Among these amino acid replacements, D111N, Q115H, E224K, and D228N were located in the NTD. Two mutations, D614G and Q675R, were in subdomains 1 and 2, and D820N, V826G, and D830N were on the S2 domain.

**Figure 2:**
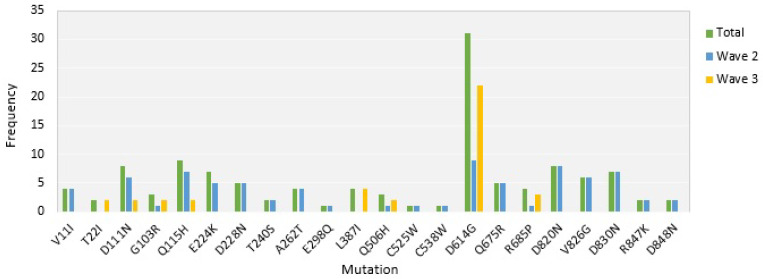
Frequency of amino acid replacements in sequences of spike glycoprotein among 60 sample sequences.

The most representative amino acid change was D614G. Its frequency was dramatically increased in patients selected from the third wave of the disease compared to patients chosen from the second wave (Figure 2).

### 3.3. Statistical Analysis

Among the nine identified nonsynonymous mutations in this study, D614G is a vital mutation that causes structural and functional changes in the SARS-CoV-2 spike protein. First, the relationship between the D614G mutation and the incidence of COVID-19 symptoms in a total population of 60 people was investigated (Table 1). Then, the relationships between sex, age, and underlying disease history with the incidence of COVID-19 clinical symptoms were assessed.

Our study revealed D614G mutation in 31 patients (51.66%). The percentage of mutated and non-mutated individuals for each clinical sign was determined and then all data were compared statistically. No significant relationship was observed for all the studied symptoms with the incidence of D614G mutation.

It has been shown that 70% (42/60) of the studied patients were male, and 30% (18/60) were female at the time of sampling. Since, in this study, the collection of samples was random, the results showed that the prevalence of COVID-19 was statistically higher in men than women. There was no significant relationship between patients’ sex and the incidence of symptoms in the study population (Supplementary Table S2).

Patients were divided into two groups according to age: ≤40 years and >40 years. After examining the relationship between symptoms and age, we found that in the studied patients, symptoms such as loss of sense of smell, headache, and chest pain were significantly higher in people ≤40 years (Supplementary Table S3).

## 4. Discussion

With the emergence of new varieties of SARS-CoV-2, controlling and combating this disease has become a global challenge [18]. The importance of the spike protein in the virulence and pathogenesis of the virus has been well identified in various studies [19]. Therefore, identifying and monitoring spike mutations and their effects on the function of the virus is essential for drug and vaccine design. Spike mutation could also directly impact spike interaction with neutralizing antibodies, leading to reinfection and even infection of fully vaccinated people [20,21].

Gender is one of the known factors in the severity of the SARS-CoV-2 disease, and men are more susceptible to the virus [22].

Different independent research has revealed that mutations in spike and other proteins of SARS-CoV-2 can potentially impact the clinical outcomes of affected patients [23,24,25]. Meta-analyses and observational studies have shown that mutations increase the risk of disease severity and death [24,25].

Therefore, different clinical outcomes may be associated with genetic mutations in SARS-CoV-2 spike glycoprotein. However, it is necessary to adjust for individual risk factors to establish such an association reliably. The most severe outcomes are expected to be associated with pre-existing diseases in affected persons. Age and comorbidities such as hypertension, obesity, cardiovascular disorders, immune suppression, smoking, and diabetes are more important predictors of severity, hospitalization, and mortality than SARS-CoV-2 mutations [26].

Some genome information of SARS-CoV-2 revealed that the mutation rate of SARS-CoV-2 is about 1∼2 nucleotides/month [26]. A similar study in Jiangsu Province of China showed that more men were infected with the SARS-CoV-2 compared to women and its tendency was to younger ages. Also, the number of asymptomatic infected patients was more (predominantly in Alpha and Beta variants), but patients infected with Delta (17%) variant presented more severe clinical features. A total of 935 SNPs were detected in 165 SARS-CoV-2 samples, and missense mutation was the dominant mutation. They found that 20% of SNP changes occurred in the spike glycoprotein (S) gene as well as nine SNPs loci in S gene were significantly correlated with the severity of disease in patients [26,27]. It is worth mentioning that amino acid substitution of p.Asp614Gly was significantly positively correlated with the clinical severity of patients. In Jiangsu Province of China, the amino acid substitution of p.Ser316Thr and p.Lu484Lys were significantly negatively correlated with the course of the disease [27].

In Turkey, results clearly showed concordance between the variant distributions, the number of cases, and the timelines of different variant accumulations [28].

D614G is a vital mutation that causes structural changes, increasing the affinity of ACE2 and leading to functional changes in SARS-CoV-2 spike protein [29]. This mutation may affect the binding activity of the S1 domain and change the secondary structure of the protein [21,27]. Zhang and colleagues [12] showed that the D614G mutation can increase the infectivity of the virus by increasing virion spike density. Becerra-Flores et al. have reported that this mutation is associated with a higher fatality rate [14]. Vanderheiden et al. computationally simulated and analyzed the functional effects of mutation D614G and found that the D614 position has a critical role in transitions of spike glycoprotein between an open and closed state [30].

In our study, D614G was the most abundant mutation in Iranian samples (Supplementary Table S1). There was no significant relationship between disease symptoms and D614G mutation in this study. D614G also is common among several of the variants of SARS-CoV-2 and increases transmissibility and viral load [13,31]. Also, in India, the spike D614G mutation has become the most common variant since December 2019. Similarly, detected in Europe and the United States, it has dramatically increased the transmission ability of SARS-CoV-2 [32]. A study has reported that high prevalence of variant D614G in the spike in Costa Rica is similar to the rest of the world. This study also showed an increased detection of a spike T1117I mutation from March to November 2020 [33]. In another study by Molina-Mora in Costa Rica, the analysis of the mutation T1117I in spike showed a polyphyletic pattern along with the emergence of local lineages around the world. This mutation seems to have a significant role in higher affinity to molecules and scarce immunity changes [34].

Our study achieved important molecular epidemiological results on the correlation of clinical features (history) with mutation of SARS-CoV-2 in the center of Iran (Isfahan province). Also, there were limitations to our study. First, this is a monocentric study. Second, some of the samples had some errors and no complete history behind them, so larger numbers of samples are further needed to minimize the errors. Also, we excluded those samples with ambiguous history. Third, the correlation of mutation with the potential biological function of variants and demographic characteristics is required for further investigations.

## Figures and Tables

**Table 1:** Statistical correlation between incidences of the clinical sings of the COVID-19 and mutation in D614G gene and their relevant *p* values.

	Incidence Rate	D614G Mutation	Wild Type	*p* Value
Total Number of Cases	60	51.66% (31/60)	48.33% (29/60)	NS
Fever	56.66% (34/60)	64.51% (20/31)	48.27% (14/29)	NS
Cough	43.33% (26/60)	51.61% (16/31)	34.48% (10/29)	NS
Muscular pain	65% (39/60)	74.19% (23/31)	55.17% (16/29)	NS
Respiratory distress	35% (21/60)	35.48% (11/31)	34.48% (10/29)	NS
Loss of consciousness	10% (6/60)	9.67% (3/31)	10.34% (3/29)	NS
Loss or less sense of smell	41.66% (25/60)	38.7% (12/31)	44.82% (13/29)	NS
Loss or less sense of taste	40% (24/60)	38.7% (12/31)	41.37% (12/29)	NS
Abdominal pain	10% (6/60)	9.67% (3/31)	10.34% (3/29)	NS
Nausea	13.33% (8/60)	19.34% (6/31)	6.89% (2/29)	NS
Vomiting	3.33% (2/60)	6.45% (2/31)	0% (0/29)	NS
Diarrhea	13.33% (8/60)	12.9% (4/31)	13.79% (4/29)	NS
Anorexia	40% (24/60)	45.16% (14/31)	34.48% (10/29)	NS
Headache	33.3% (20/60)	38.7% (12/31)	27.58% (8/29)	NS
Dizziness	26.6% (16/60)	29.03% (9/31)	24.13% (7/29)	NS
Chest pain	16.66% (10/60)	12.9% (4/31)	20.68% (6/29)	NS
Inflammation or skin lesion	5% (3/60)	3.22% (1/31)	6.89% (2/29)	NS

*p* < 0.05 = significant difference; NS = Non-significant (*p* > 0.05); 0.05 > *p* > 0.10 = tendency.

## Data Availability

The data of this research would be available upon request to corresponding author, subjected to confirmation of Ethical Board Committee.

## Supplementary Materials

The following supporting information can be downloaded at: https://www.mdpi.com/article/10.3390/jcm11216315/s1, Table S1. Nonsynonymous mutations identified in the spike protein of SARS-CoV-2 isolated from COVID-19 patients in Isfahan, Iran compared with hCoV-19/Wuhan/WIV04/2019; Table S2. Association between patients’ sex and COVID-19 symptoms in patients; Table S3. Association between age and COVID-19 symptoms in patients.
