# Supplementary material for: Association of Clinical Features with Spike Glycoprotein Mutations in Iranian COVID-19 Patients
Source: J Clin Med. 2022 Oct 26;11(21):6315. doi: 10.3390/jcm11216315 (PMC9654899; doi:10.3390/jcm11216315)
Supplement: Supplementary file 1 [file jcm-11-06315-s001.zip › jcm-1950879-supplementary.pdf]

Supplementary Materials

Table S1. Nonsynonymous mutations identified in spike protein of SARS-CoV-2 isolated from COVID-19 patients in Isfahan, Iran compared with hCoV-19/Wuhan/WIV04/ 2019.

Mutations	Total No. (n = 60)	Wave 2	Wave 3	NCBI Accession ID of Submitted Sequence Where the Mutation is Detected
D111N	8	6	2	MW132940.1(54), MW113237.1(61), MW113670.1 (62), MW114306.1 (63), MW132928.1 (65), MW114446.1 (69), MZ206020.1 (85), MZ206163.1 (91)
Q115H	9	7	2	MW322566.1 (50), MW113237.1(61), MW113670.1 (62), MW114306.1 (63), MW114523.1(64), MW114305.1 (66), MZ203839.1 (71), MZ206020.1 (85), MZ206163.1 (91)
E224K	7	5	0	MW126756.1(22), MW320670.1 (25), MW321439.1(44), MW321481.1 (46), MW321483.1 (47), MW321496.1 (49), MW132896.1(56)
D228N	5	5	0	MW320670.1 (25), MW321439.1(44), MW321481.1 (46), MW321496.1 (49), MW132896.1(56)
D614G	31	9	22	MW126756.1 (22), MW320672.1 (28), MW321483.1 (47), MW321496.1 (49), MW113237.1 (61), MW132928.1 (65), MW114305.1 (66), MW113679.1 (68), MW114446.1 (69), MZ203839.1 (71), MZ203840.1 (72), MZ203841.1 (73), MZ203843.1 (75), MZ203857.1 (77), MZ203865.1 (81), MZ206017.1 (83), MZ206018.1 (84), MZ206020.1(85), MZ206021.1 (86), MZ206131.1 (87), MZ206132.1 (88), MZ206133.1 (89), MZ206163.1 (91), MZ206164.1 (92), MZ206168.1 (95), MZ206170.1 (96), MZ206169.1 (97), MZ882185.1 (102), MZ882187.1 (103), MZ882256.1 (104), MZ882258.1 (106)
Q675R	5	5	0	MW321436.1 (42), MW321481.1 (46), MW132940.1(54), MW113670.1 (62), MW114306.1 (63)
D820N	8	8	0	MW320670.1 (25), MW320673.1 (27), MW320672.1 (28), MW320721.1 (38), MW321439.1(44), MW321481.1 (46), MW321496.1 (49), MW132896.1(56)
V826G	6	6	0	MW126756.1(22), MW320670.1 (25), MW320671.1 (26), MW320673.1 (27), MW321481.1 (46), MW321496.1 (49)
D830N	7	7	0	MW320670.1 (25), MW320671.1 (26), MW320673.1 (27), MW321439.1(44), MW321496.1 (49), MW322566.1 (50), MW132896.1(56)

Table S2. Association between patients' sex and COVID-19 symptoms in patients.

	Incidence rate	Female	Male	p Value
**Total Number of Case**	60	30% (18/60)	70% (42/60)	**<0.001**
**Fever**	56.66% (34/60)	50% (9/18)	59.52% (25/42)	NS
**Cough**	43.33% (26/60)	38.88% (7/18)	45.23% (19/42)	NS
**Muscular Pain**	65% (39/60)	72.22% (13/18)	61.90% (26/42)	NS
**Respiratory distress**	35% (21/60)	22.22% (4/18)	40.47% (17/42)	NS
**Loss of consciousness**	10% (6/60)	5.55% (1/18)	11.90% (5/42)	NS
**Loss or less sense of Smell**	41.83% (25/60)	44.44% (8/18)	40.47% (17/42)	NS
**Loss or less sense of taste**	40% (24/60)	38.88% (7/18)	40.47% (17/42)	NS
**Nausea**	13.33% (8/60)	11.11% (2/18)	14.28% (6/42)	NS
**Vomiting**	3.3% (2/60)	5.55% (1/18)	2.38% (1/42)	NS
**Diarrhea**	13.33% (8/60)	16.66% (3/18)	11.90% (5/42)	NS
**Anorexia**	40% (24/60)	38.88% (7/18)	40.47% (17/42)	NS
**Headache**	33.33% (20/60)	38.88% (7/18)	30.95% (13/42)	NS
**Dizziness**	26.6% (16/60)	27.27% (5/18)	26.19% (11/42)	NS
**Chest pain**	16.66% (10/60)	16.66% (3/18)	16.66% (7/42)	NS

**Inflammation or skin lesion**	5% (3/60)	0.00% (0/18)	7.14% (3/42)	NS

$p < 0.05$ = significant difference (bold); NS = Non-significant ($p > 0.05$); $0.05 > p > 0.10$ = tendency.

Table S3. Association between age and COVID-19 symptoms in patients.

	**Incidence Rate**	**≤40 Year**	**>40 Year**	***p* Value**
**Total Number of Case**	60	31.66% (19/60)	68.33% (41/60)	**<0.001**
**Fever**	56.66% (34/60)	52.63% (10/19)	58.53% (24/41)	NS
**Cough**	43.33% (26/60)	47.36% (9/19)	41.46% (17/41)	NS
**Muscular Pain**	65% (39/60)	78.94% (15/19)	82.92% (34/41)	NS
**Respiratory distress**	35% (21/60)	47.36% (9/19)	29.26% (12/41)	NS
**Loss of consciousness**	10% (6/60)	10.52% (2/19)	9.75% (4/41)	NS
**Loss or less sense of Smell**	41.83% (25/60)	68.42% (13/19)	29.26% (12/41)	**0.006**
**Loss or less sense of taste**	40% (24/60)	57.89% (11/19)	31.70% (13/41)	0.058
**Nausea**	13.33% (8/60)	15.78% (3/19)	12.19% (5/41)	NS
**Vomiting**	3.3% (2/60)	10.52% (2/19)	0.00% (0/41)	NS
**Diarrhea**	13.33% (8/60)	21.05% (4/19)	9.75% (4/41)	NS
**Anorexia**	40% (24/60)	47.36% (9/19)	36.58% (15/41)	NS
**Headache**	33.33% (20/60)	52.63% (10/19)	24.36% (10/41)	**0.034**
**Dizziness**	26.6% (16/60)	42.10% (8/19)	19.51% (8/41)	**0.071**
**Chest pain**	16.66% (10/60)	31.54% (6/18)	9.75% (4/41)	**0.044**
**Inflammation or skin lesion**	5% (3/60)	5.26% (1/19)	4.87% (2/41)	NS

$p < 0.05$ = significant difference (bold); NS = Non-significant ($p > 0.05$); $0.05 > p > 0.10$ = tendency.
